# Enteric Fever in Cambodia: Community Perceptions and Practices Concerning Disease Transmission and Treatment

**DOI:** 10.4269/ajtmh.18-0432

**Published:** 2018-10-16

**Authors:** Laura Maria Francisca Kuijpers, Charlotte Gryseels, Sambunny Uk, Panha Chung, Sotharith Bory, Bun Sreng, Amy Parry, Jan Jacobs, Koen Peeters Grietens

**Affiliations:** 1 Department of Clinical Sciences, Institute of Tropical Medicine, Antwerp, Belgium;; 2 Department of Microbiology and Immunology, KU Leuven, Leuven, Belgium;; 3 Department of Public Health, Institute of Tropical Medicine, Antwerp, Belgium;; 4 Independent Researcher, Phnom Penh, Cambodia;; 5 Sihanouk Hospital Center of HOPE, Phnom Penh, Cambodia;; 6 Calmette Hospital, Phnom Penh, Cambodia;; 7 Department of Communicable Disease Control, Ministry of Health, Phnom Penh, Cambodia

## Abstract

Enteric fever is a systemic bacterial infection in humans that is endemic in Cambodia and for which antibiotic resistance is increasingly reported. To guide public health programs, this qualitative study sought to explore community perceptions on transmission and treatment. Participant observation was carried out in hospital settings, pharmacies, and at a community level in Phnom Penh. In-depth interviews 39 and one focus group discussion were carried out with blood culture–confirmed enteric fever patients and purposively selected key informants. Informants were theoretically sampled based on initial themes identified using abductive analysis. Nvivo 11 was used for thematic coding. An urgent need to address health literacy concerning the transmission of enteric fever was identified, as lay informants did not link the disease and its symptoms to bacterial contamination of foods and drinks but rather to foods considered “bad” following humoral illness interpretations. As a result, lay informants considered recurrence of enteric fever preventable with appropriate dietary restrictions and Khmer traditional medicines. This study also reveals pluralistic health-care–seeking behavior. For initial and mild symptoms, patients preferred home treatment or traditional healing practices; limited household finances delayed treatment seeking. When symptoms persisted, patients first visited drug outlets or private practitioners, where they received a mix of nonessential medicines and one or more antibiotics often without prescription or confirmation of diagnosis. Inappropriate use of antibiotics was common and was related to diagnostic uncertainty and limited finances, factors which should be addressed during future efforts to improve the uptake of appropriate diagnostics and treatment of enteric fever.

## INTRODUCTION

*Salmonella enterica* serovars Typhi (*Salmonella* Typhi) and Paratyphi (*Salmonella* Paratyphi) A, B, and C are bacteria that cause typhoid and paratyphoid fever, respectively (also jointly referred to as “enteric fever”). Humans are the only host to these bacteria and the central mechanism by which the disease is transmitted is fecal shedding of the bacterium after infection, also known as carriage. Carriage is asymptomatic and can be either temporary (through intestinal lesions) or chronic (through gallbladder colonization). In countries with poor sanitation and food hygiene, the bacteria can contaminate water and food items. When a sufficient number of bacteria are ingested, they reach and invade the small intestine and eventually the blood circulation.^1^ At this stage, patients typically experience persistent fever and other nonspecific symptoms.^2^ The diagnosis is confirmed by culturing the causative bacterium from blood, which requires microbiology laboratories that are often absent in endemic settings. In addition, the sensitivity of blood cultures is poor (40–80%) and diagnosis can take up to 2–3 days.^3,4^ Often, health-care workers will resort to clinically unreliable but faster and cheaper alternative diagnostic methods (e.g., Widal test that detects *Salmonella* antibodies in blood). Confirmed cases, therefore, represent only a small proportion of the total affected population.

Enteric fever can result in life-threatening complications, such as intestinal perforation and gastro-intestinal bleeding.^5^ Before the introduction of antibiotics, enteric fever carried a case fatality rate of 10–30% compared with 1% when effective antibiotics are used.^6^ If noneffective or no antibiotics are used, the disease is estimated to relapse in 5–10% of confirmed cases.^5^

Enteric fever is highly endemic in rural and urban Cambodia.^2,7,8^ During 2012–2015, the capital city of Phnom Penh experienced a community outbreak of *Salmonella* Paratyphi A infections, affecting large numbers of local residents and international travelers.^9–12^ Despite epidemiological investigations by public health authorities, the source of the outbreak remained unknown.^10^ In addition, little was known about local challenges in relation to disease prevention, diagnosis, and treatment. A hospital in Phnom Penh recently reported an increase in resistance against ciprofloxacin, an affordable antibiotic and the current first-line treatment.^2^ The development and spread of resistance is likely a result of the frequently cited irrational use of antibiotics in Cambodia.^13–15^ Over-the-counter sale of antibiotics from community pharmacies is common and linked to poverty, limited access to (quality) diagnostics and a lack of (enforcement of) regulations.^16–18^

Irrational antibiotic use and disease transmission are known to be facilitated and even driven by human behavioral factors^19–21^ and structural constraints.^22^ Knowledge and cultural interpretations of illness, as well as (lack of) access to affordable good quality care and diagnosis, affect exposure to the disease and patients’ health-care–seeking and therapy choice. A more in-depth understanding of these factors is essential for effective public health inventions aiming to interrupt the transmission cycle and to contain the spread of antibiotic resistance. The primary objectives of this study were, among both laypersons and health-care providers in Phnom Penh, to 1) assess illness interpretations (causes, risks, and transmission); 2) explore perceptions on treatment; and 3) identify first-line treatment practices and health-seeking behavior associated with enteric fever.

## METHODS

### Research design.

Qualitative data were collected through ethnographic research techniques, namely participant observation, semi-structured interviews, informal conversations, focus group discussions (FGD), and a review of written documents and webpages (English and Khmer). Data were triangulated to limit response bias, and to complement and strengthen the validity of the data collected by the respective research techniques.

### Study site and population.

This study was conducted between September 2015 and April 2017 in Phnom Penh, the capital city of Cambodia, a low-middle income country located in Southeast Asia. Cambodia’s health-care system collapsed during Pol Pot’s regimen (1975–1979), when large numbers of health-care providers either left the country or were killed while health facilities were destroyed.^23,24^ During the last three decades the health system was rebuilt and the country is currently experiencing impressive economic growth. However, poverty and communicable diseases remain common.^25^ Cambodia has one of the highest shares of out-of-pocket payments for health care in the region; the majority (68%) go to private medical services.^26^ An estimated 1.55 million people live in Phnom Penh, of which approximately 82% has access to sanitation facilities such as a piped sewer system and a septic tank.^27^ Cambodia has a “tropical monsoon climate comprising annual dry and wet seasons.”^27^ Most enteric fever cases were reported during the dry season.^2^

Enteric fever cases were defined as patients with a blood culture positive with either *Salmonella* Typhi or *Salmonella* Paratyphi A.

### Concept definitions.

1) *Enteric fever* is used with the limitation that its translation in Khmer, “*krun pos vean*” (fever of the intestines), is also used to describe clinically unconfirmed cases, which may include other abdominal illnesses with similar symptoms. 2) *Drug sellers* refer to both licensed pharmacists and unlicensed drug sellers due to difficulties in differentiating them. 3) *Physicians* indicate medical doctors working in a health-care facility, such as a public hospital or a private clinic. 4) *Private practitioners* are considered medical doctors who primarily work in a private consultation room, often located within their own residence. Some have received specialty training whereas others practice general medicine. Many private practitioners also work in a public hospital and keep supplies of medicine to sell to their patients.

### Sampling.

Sampling was theoretical, meaning that participants were chosen purposively based on preliminary results and in line with the theory emerging from the data. Blood culture–confirmed enteric fever patients were recruited at the Sihanouk Hospital Center of HOPE (SHCH), a nongovernmental referral hospital in Phnom Penh. Sihanouk Hospital Center of HOPE and its associated clinics provide care with reduced fees for the poor. This hospital was selected based on its involvement in a blood culture surveillance program, allowing the recruitment of confirmed cases. All patients with a blood culture positive with *Salmonella* Typhi or *Salmonella* Paratyphi A between August 2015 and April 2017 were contacted by phone by SHCH staff. Participants include both confirmed patients and other key informants, comprising drug sellers, physicians, nurses, laboratory staff, Khmer medicine sellers, and farmers. Key informants were used to identify additional participants for snowball sampling. Sampling continued until data saturation was reached.

### Data collection.

Participant observation consisted of visiting patients’ homes, pharmacies/drug outlets, clinics, private consultation rooms, and Khmer medicine shops and markets. These observations offered the additional opportunity for a number of informal conversations with other community members. During visits to patients’ homes, living conditions, antibiotic prescriptions, and medicine purchases were observed.

A semi-structured and informal interview approach with open questions was used. Interview guides were inspired by initial hypotheses and a question guide based on a previously developed questionnaire implemented during a case–control study on enteric fever in Indonesia.^28^ Guides were continuously adapted as new insights emerged. Whenever possible, interviews were recorded and transcribed and, when necessary, translated into English. When not possible and/or inappropriate, the content of the conversation was written down during the interview or immediately afterward. Interviews were carried out by a local researcher (SU) and/or by a physician (LMFK), both trained in qualitative research methods.

One FGD was conducted with physicians from a private medical clinic.

As part of the triangulation process, text analysis of written documents, such as newspaper articles and website pages, was continuously carried out, leading to the identification of emergent themes and informants.

### Data analysis.

Data analysis was an iterative and abductive process. The PASS health seeking behavior model (developed within the PASS International organization) was used to guide the initial analytic process.^29^ Transcripts, memos, and field notes were continuously analyzed for thematic content and emerging hypotheses and results were tested in the field until saturation was reached. Thematic analysis of all data was carried out using NVivo 11 qualitative analysis software (QRS International Pty., Cardigan, United Kingdom).

### Ethical clearance.

This study was approved by the institutional review board of the Institute of Tropical Medicine in Antwerp, Belgium (ref. 1001/15, dated April 8, 2015) and the National Ethics Committee for Health Research (NECHR) in Cambodia (ref. 216 NECHR, dated June 29, 2015, and ref. 254 NECHR dated June 27, 2016). The interviewers followed the Code of Ethics of the American Anthropological Association. Participants were informed about project goals, the topic and type of questions as well as their right to decline participation or to interrupt the conversation at any time. Confidentiality of interviewees was assured by assigning a unique code number to each informant. Verbal consent, approved by both ethics committees, was preferred to encourage an open expression of opinions.

## RESULTS

### Participants.

In total, 39 in-depth interviews were conducted, 21 of which were carried out with blood culture–confirmed enteric fever patients (Table 1) and 18 with additional key informants.

**Table 1:** Overview of participants by data collection technique

Participants	Interviews (*N* = 39)	Informal conversations (*N* = 38)	Observations (*N* = 13)
Lay persons			
*Salmonella* Paratyphi A patient	20	0	3
*Salmonella* Typhi patient	1	0	–
Suspected enteric fever case*	2	0	–
Relatives/friends of patients	0	2	–
Traditional medicine producers/sellers	0	7	–
Khmer traditional healer	0	1	–
Farmers	–	4	3
Food vendors	1	1	3
Other	–	2	1
Health-care workers			
Physicians/private practitioners	8	8	–
Nurses	3	0	–
Laboratory staff		5	–
Drug sellers	4	10	1

* Suspected enteric fever case refers to those patients who had received a diagnosis of enteric fever, which had not been confirmed with blood culture.

In total, 39 patients were diagnosed with enteric fever during the study period, 35 of which were reached by phone; 31 agreed to be interviewed. Reasons stated for declining included limited time available and residence outside of Phnom Penh. Some expressed fear of being interviewed, meaning fear of losing face or being found to have done something wrong.

Ten patients agreed to participate but could not be interviewed as they did not return to the hospital for treatment and were reportedly engaged in work or family obligations. The median age of patients interviewed was 26 (range 20–62), nine (42.9%) were male. Patients resided in eight different districts of Phnom Penh. One patient reported an address outside of the city. Interviews were conducted at the clinic or at patients’ homes. Eighteen interviews were conducted with physicians, private practitioners, and drug sellers. Thirty-eight informal conversations were held with the sellers of biomedical and traditional medicines, laboratory staff, patients’ relatives, and farmers. One FGD was conducted with five physicians and one laboratory staff from a private clinic.

### Humoral illness interpretations.

In Khmer, enteric fever translates as “*krun pos vean*”, literally meaning “fever of the intestines,” a term widely known and frequently used by patients and key informants to indicate episodes of fever and/or abdominal complaints. Lay informants described the illness as a compilation of different illness symptoms, including stomach ache and diarrhea, rather than as a single disease with a single cause. They perceived symptoms to be consequences of the ingestion of “bad foods,” where “bad” was defined following culturally defined humoral illness interpretations. This humoral system categorizes illnesses and possible causes according to their “hot” and “cold” qualities, whereby good health requires a balance between both. Food and drinks, such as rice and coffee, and specific activities (such as working hard) are classified as hot or cold. Examples of hot foods are those with a sour flavor such as guava and green mango, or spiced foods, such as instant noodles and coffee.

Lay informants associated the excess consumption of these “hot” foods with certain processes inside the body that explained the occurrence of specific symptoms associated with enteric fever, such as obstipation, fever and abdominal pain. As an example, the ingestion of sour food was associated with irritation of the intestinal walls and consequent constipation. To counteract these symptoms, patients were generally advised by family and friends to avoid particular foods and drinks. For example, eating rice was discouraged as it was considered to damage the weakened intestinal walls and to generate heat. Rice soup was recommended instead.

These dietary restrictions were also considered important for the prevention of “*loarp*”, a local illness category that refers to perceived relapses of previous illness episodes. *Loarp* episodes of enteric fever are perceived to be caused by an imbalance in the humors of the body due to hot foods or physical activities during recovery.

### “Mae rhouk” and risk perceptions.

The Khmer language does not have separate words for bacteria and viruses. Both are translated as “*mae rhouk*”. Sometimes physicians used “*bacterie*” or “*mae rhouk-bacterie*” to differentiate a bacterium from a virus, but this differentiation was not made by lay people. As mentioned previously, lay informants associated the cause of *mae rhouk* illnesses with the consumption of “bad foods.” Additional causes were considered to be “bad hygiene” and eating meals outside, although how this could to lead to enteric fever was often unclear. Having meals at irregular times was associated with stomachaches due to gastric acid in an empty stomach.

The most frequently mentioned concrete vehicle for transmission of enteric fever by participants were clams (*leas harl*) and snails, which are popular and affordable snacks collected from river shores in the countryside and sold throughout the city on mobile carts. Especially clams were associated with enteric fever, as well as diarrhea and parasitic infections, mainly because of their proximity to sand, which was perceived to equal dirt. Increased sales of clams, easier to catch when water levels in rivers are low, were associated with the dry season. In addition, during this season clams are dried in the sunlight because boiling is considered to shrink the meat and to spoil the taste.

Consumption of (raw) vegetables was also often mentioned to be a risk factor for enteric fever. Some key informants linked this to the practice of fertilizing crops with human stool whereas others referred to the vegetables grown on Phnom Penh’s “contaminated” peri-urban lakes. Observations at these lakes showed sewage pipes and toilets emptying directly into the water close to where several types of popular vegetables (such as Morning glory and *Kan Chet*) and herbs (*ghee*) were cultivated.

Perceived prevalence of the disease was thought to vary substantially according to the season and the particularities of each season to be risk factors. The dry season was associated with harvesting vegetables and with higher temperatures and an associated indiscriminate consumption of water and ice cubes. Small ice cubes were referred to as “*hygiene ice*” even when the water source was unknown. Participants also observed longer food storage times among street vendors and increased demands during festivities in the dry season. The rainy season was associated with flooding, thought to increase the risk of enteric fever and other diarrheal illnesses, and with the increased availability of fruit and the subsequent presence of flies, which were considered to be potential disease vectors.

### Humoral interpretations and treatment choice.

Humoral interpretations of illness guided initial treatment choice. Depending on specific symptoms, common home remedies such as ingestion of ginger or massage techniques such as coining were used (i.e., a coin is used to stroke the skin firmly resulting in red colored bands which is interpreted as released heat). These therapies were only used when symptoms were considered mild. Biomedical pharmaceuticals such as paracetamol bought from local pharmacies were also popular as home remedies for mild symptoms. Patients with enteric fever reported to use different types of home remedies and to wait a few days before seeking treatment outside the home.

**Figure 1.**
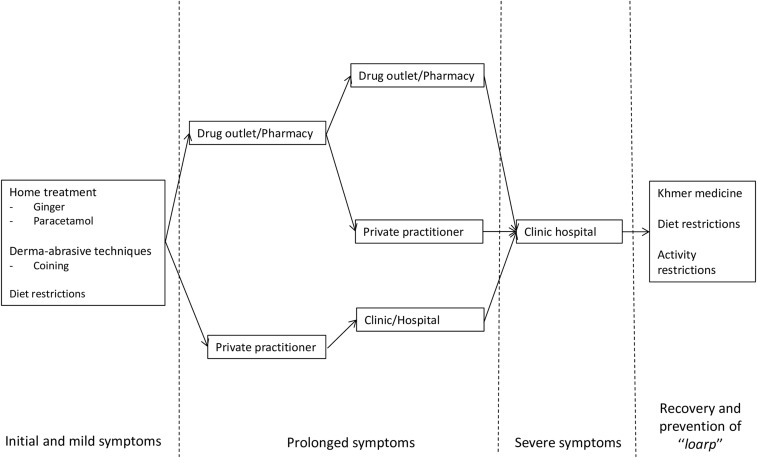
Health seeking behavior by patients with symptoms suggestive of enteric fever.

If symptoms did not improve after approximately 2–3 days, most patients reported to visit a pharmacy or drug outlet to buy a cocktail of medicines. Only few consulted a private practitioner or went to a hospital directly. The reason for first visiting a drug seller rather than a doctor was mostly related to the perceived lower cost. When the fever and/or other symptoms did not improve after taking the received medicines two to three times, the patient readily switched to a different pharmacy, drug outlet, and private practitioner or clinic. If symptoms persisted or became more severe, patients eventually visited the hospital (Figure 1). The choice for a particular clinic or health-care provider was based on 1) recommendations of others; 2) previous experiences; 3) relatives that worked at the facility; 4) availability of health insurance policy; 5) reduced costs for the poor; and 6) presence of “good electronics.”

Most drug sellers and private practitioners mentioned that they advised patients to get a blood test (Widal test) in case of suspected enteric fever but that this would often be refused. Stool cultures were requested even less frequently.

*I bought mixed medicines on Thursday. I normally take medicine by myself such as Spasfon [antispasmodic] to help me with abdominal pain, but this time I had to buy mixed medicines from the pharmacy. I was told by the drug seller that since I had a fever for a longer time, I should go to get a blood test at a hospital. I asked them to just sell me 3 swallows of mixed medicines.* (Enteric fever patient, female)

Drug sellers reported that only approximately one in 10 patients attended with a doctor’s prescription. Laboratory tests were seldom carried out, and if they were, seldom acted on due to the additional cost for the patient, logistical challenges, limited confidence in laboratories, and the perception that the diagnostic test would not influence the choice of treatment.

*I give the same treatment for food poisoning and infectious diarrhea, so in fact there is no point in further confirming a diagnosis, the treatment is the same.* (Private practitioner, female)

Although Khmer traditional medicine shops were also visited in the initial stages of the illness, herbal medicines were mostly used after patients had already completed biomedical treatment. For enteric fever–associated symptoms, Khmer medicine shops offered a range of products including pills (e.g., consisting of bitter or “cooling” vegetables and leafs such as “*Sdav*” and “*Sleouk malou*”), bottles with liquid concoctions (gourd leaf with bitter melon or coconut juice with charcoal) or roots to make tea. These types of treatments were considered especially potent for the prevention of *loarp* episodes.

### “Thnam psom”: mixed medicine.

For both public and private medical practitioners, it was common to treat each symptom with a different drug. The aim was the fast resolution of each symptom and thereby satisfying the patient’s expectations. If symptoms did not subside quickly, patients readily switched to another health-care provider.

*With real typhoid, the increase in temperature goes step by step and also decreases slowly, not like with dengue fever. That’s why you have to give an anti-pyretic drug, to make the temperature go down faster, otherwise the patients will go away. When the symptoms go away fast, the people think: that doctor is smart.* (Private practitioner, male)

**Figure 2.**
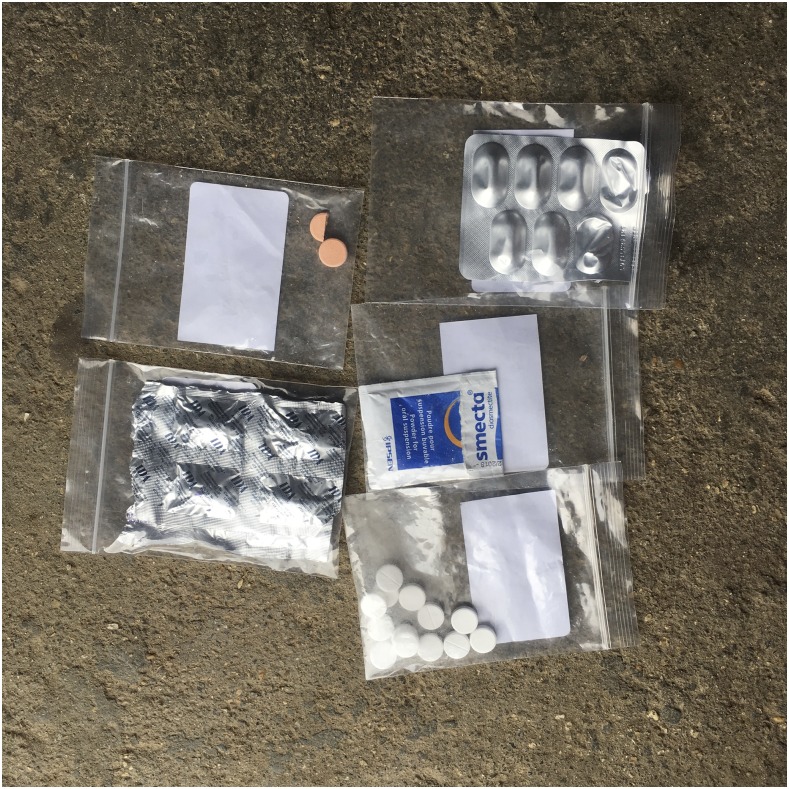
“Mixed medicine” taken at home by a confirmed enteric fever patient. This figure appears in color at www.ajtmh.org.

Treating individual symptoms required a mix of different medicines, generally including a combination of: 1) paracetamol, 2) antidiarrheals, 3) one or two antibiotics, 4) vitamins, 5) anticold or cough medicine, 6) antacids, and 7) anti-inflammatory medicines or steroids (Figure 2). At drug outlets, these pills were often removed from blisters and prepackaged in single plastic bags. Also physicians frequently prescribed combinations of at least five different medicines. These mixed medicine bags were referred to as “*thnam psom*”, or “unknown medicines” because patients were usually not aware what specific medicines they have received.

### “Thnam psaas”: the drug that heals.

The cocktails of medicines patients received often included antibiotics, commonly referred to as “*thnam psaas*” in Khmer, which translates as the “drug that heals.” Some physicians preferred to use the word “*thnam samlap mae rhouk*” instead, which literally translates as “drug that kills pathogen.” Customers often knew and requested certain antibiotics by name.

Physicians and drug vendors reported ciprofloxacin, or other antibiotics of the fluoroquinolone group, to be the number one antibiotic prescribed and/or dispensed for suspected enteric fever. This group of antibiotics was considered effective, cheap, and beneficial for a number of other conditions. With a few exceptions, health-care workers generally were unaware that decreased susceptibility to ciprofloxacin was now common and even those that were aware continued prescribing it:

*I treat [enteric fever] with ciprofloxacin. I know there is resistance but I give it anyway. People don’t want to pay for the expensive medicines or the injections. I tell them to see a doctor if after 3 days, they don’t feel better.* (Expat physician, male)

Both health-care workers and laypersons perceived the efficacy of antibiotics to be associated with the brand and/or country of manufacturing. The choice for a certain antibiotic brand normally depended on the amount of money the patient was willing to spend, the more expensive brands being considered superior in quality. French antibiotics were considered to be the most potent. In addition, drug sellers’ choice of antibiotic(s) depended on the perceived severity of disease, the availability of a laboratory result and own experience, rather than treatment guidelines.

Ofloxacin* or ciprofloxacin can be combined with co-trimoxazole, cimetidine

[inhibits stomach acid production], Spasfon [antispasmodic] and paracetamol.

*The patients get an injection in my house if they have a laboratory result, if they don’t have a laboratory result, I never inject antibiotics to them*…

*When the enteric fever is severe with the result of the laboratory they are injected [ceftriaxone] 2 times a day plus gentamycin... While I use ceftri [ceftriaxone] and genta [gentamycin], I don’t use cipro [ciprofloxacin]. … If they didn’t get a laboratory test they are prescribed ciprofloxacin.* (Drug seller, male)

Drug sellers reported that patients rarely purchased a full course of medicines. They acknowledged this as a problem but could not refuse to sell small quantities as they feared loss of clientele. Customers generally purchased the quantity of pills that they could afford. Treatment adherence was also affected by the perceived negative effects of medicines. “Western” medicines, referring to antibiotics and other types of biomedical treatment, were believed to have a dual effect on the body: they could help cure a diseased organ, but at the same time, negatively impact other, healthy, organs. Patients and key informants explained that if taken for too long, or too many times, Western medicines such as antibiotics could cause “new illnesses,” referring to diabetes, stomach disease, or fungal infections. In the case of antibiotics, a variety of other medicines (such as stomach protectors) were perceived to be needed to counter the “strong” effect antibiotics have on other organs, in particular the stomach.

Physicians at SHCH stated that most of their patients returned for daily injections of ceftriaxone, the first-line antibiotic treatment of complicated enteric fever, often completing 10–14 days of treatment. This was attributed to the time spent explaining to patients the benefits of completing treatment even after resolution of symptoms.

## DISCUSSION

This study explored local illness interpretations, perceptions on transmission and treatment of enteric fever and associated practices in Phnom Penh, Cambodia, during the aftermath of a *Salmonella* Paratyphi A outbreak. In line with previous ethnographic studies from Asia,^30,31^ our study shows that lay persons perceived symptoms associated with enteric fever to be consequences of ingesting “bad foods,” where the concept of “bad” was grounded in humoral interpretations of health and illness.^32,33^ Bacterial contamination of food and water was not perceived as a direct cause of the disease by patients, suggesting limited awareness of the key role food hygiene and sanitation play in transmitting and preventing enteric fever.

The nonspecific and initial mild symptoms and limited household finances resulted in a delay of seeking care at a health-care facility. Patients were often treated at drug outlets instead, without a microbiological confirmation of the diagnosis and with a cocktail of medicines prescribed for a limited duration of time. As a result, the small amount of money the average patient had, was spread over a few nonessential medicines rather than on a full course of antibiotics.^13,15^ Home use of pharmaceuticals, such as paracetamol, or more traditional remedies, such as ginger, were common early responses to illness symptoms.

If symptoms persisted after a first health provider visit, patients readily switched among and between drug sellers, private practitioners, public health facility doctors, and/or traditional healers. This shows the complexity of the current pluralistic health-care system in Cambodia where providers from different backgrounds and sectors compete over clients. Drug sellers and private practitioners described patient pressure to sell small quantities of antibiotics, preferably without the need of confirming the disease using laboratory tests.^13,34^ This observed preference for a short duration of antibiotic therapy seems to be guided by economic motives and fears of negative effects, that is, side effects, of “Western medicines.” Often drug sellers gave in to this pressure for the fear of loss of clientele. In line with a previous qualitative study among Cambodian physicians,^14^ antibiotic prescribing was facilitated by diagnostic uncertainty, as laboratory tests were rarely performed due to financial constraints of clients, logistical challenges, and limited confidence in laboratories. The reported high percentage of nonprescribed sales of antibiotics is worrisome, given its association with short courses and inappropriate drug and dose choice, and subsequently, treatment failure and the development of antibiotic resistance.^17,21^ Economic motives and drug seller’s personal experience, rather than locally defined susceptibility patterns, guided antibiotic selection. Prescribing combinations of different antibiotics, which was observed to be common practice, currently has no place in official treatment guidelines for enteric fever.

Surprisingly, some participants discussed the use of traditional Khmer medicine at early or late stages of an enteric fever episode. Khmer medicine consists of a mix of medical beliefs and practices adapted from Chinese medicine, Ayurvedic medicine and French pharmaceutical practices.^32^ Popular in Khmer medicine are massage techniques such as coining which are thought to restore balance by releasing air and/or heat, associated with disease symptoms, from the body.^32^ Khmer traditional medicine was thought to be particularly effective in preventing a condition called *loarp*, associated with a relapse of symptoms and which was generally feared. In biomedical terms relapse occurs when the prescribed antibiotics are ineffective or when the treatment duration is too short. The local interpretation of *loarp*, however, includes working hard and eating hot or hard food as examples of perceived precipitating factors, following humoral theory rather than a biomedical explanatory model. A recent study from Nepal also found that enteric fever patients were concerned about the recurrence of the disease and used supplements and special diets to prevent this.^35^ The concerns and awareness regarding relapse or recurrence suggest that it perhaps is more common in the community than the 5–10% hospital-based estimate.^5^

Although not designed for this purpose, the present study identified several practices that could be associated with an increased risk of enteric fever. Some of the raw vegetables and herbs used in popular street food were found to be cultivated in Phnom Penh’s peri-urban lakes, known to receive 80% of the city’s untreated wastewater.^36^ Indeed, vegetables cultivated in these lakes were previously found to be highly contaminated with faeces.^37^ Case–control studies on typhoid fever have identified consumption of raw vegetables as a risk factor for the disease,^38,39^ and a recent community outbreak of paratyphoid fever in China was traced back to crops irrigated with waste water.^40^ Street food and snacks sold by vendors and hawkers in public places have also been linked to increased risk of enteric fever.^28,41,42^ In Phnom Penh, clams are popular snacks sold on the streets and our study found they are often not boiled during the dry season as this was thought to spoil the taste. Shell fish are known to be able to concentrate microorganisms such as *Salmonella* from water; a previous paratyphoid fever outbreak in Singapore was linked to contaminated oysters.^43^

The aforementioned findings have several implications. First, the contextual information regarding health-care–seeking behavior and delays in health-care access can be used to improve current surveillance activities.^35,44^ Disease surveillance is “essential for characterizing disease burden, defining public health priorities, monitoring, and evaluating disease prevention programs and identifying outbreaks.”^45^ Surveillance for infectious diseases is focused on health-care facilities and therefore does not capture patients who seek care elsewhere.^46^ As health-care–seeking behavior varies between settings and by illness, information on these patterns helps estimate the true burden of disease.

Second, the findings underline the need for increased health literacy regarding enteric fever in the population and, in particular, on the role that food and personal hygiene play in preventing transmission. During a typhoid fever outbreak in Malawi, “typhoid talks,” whereby lay people were informed on the causes, treatment, and prevention of typhoid fever, were held during community meetings.^47^ These health programs can be more effective when they build upon preexisting and culturally defined concepts of disease causation and transmission.^48–50^ In Cambodia, programs could also focus on discouraging growing vegetables on and along heavily contaminated peri-urban lakes and relocating farmers from these lakes to other locations.^51^

Third, education campaigns could stress the importance of a full course treatment and provide accurate information on potential side effects of antibiotics. The observed fear regarding recurrence of disease symptoms could prove a powerful entry point for encouraging uptake of full courses of antibiotics.

Last, the present study shows that limited household finances impede access to formal health care, diagnostics and appropriate therapy. This is despite the several policies that the Cambodian government has put in place, including user fee exemptions and health equity funds.^52^ The development and implementation of the National Social Security Fund and envisaged social health insurance will be a good step toward reducing the current high share of out-of-pocket payments and providing better access to health care.^26,52^ The urgency for these measures is demonstrated by the multiple reports on increasing antibiotic resistance in Cambodia.^2,53,54^ In response to the rising resistance rates, a newly formed antimicrobial resistance technical working group in Cambodia will address some of the identified causes in coming years.

### Study limitations.

We found that clinical diagnosis and the Widal test are more commonly used than blood cultures, so blood culture–confirmed patients represent only a small proportion of the total population affected. For this reason, we also interviewed suspected enteric fever cases and other lay informants. Patients were recruited at one center only, but this center comprised several clinics spread across the city, some of which had reduced rates for the poor and/or free provision of blood cultures, leading to socioeconomic and geographic variation in the sample. One-third of all confirmed patients, however, could not be interviewed because of logistical constraints or fear of being interviewed, which might introduce some selection bias. The study was conducted in Phnom Penh and is, therefore, not representative for rural communities. Some people from rural communities do seek health care in Phnom Penh, probably those who can afford to travel and stay in Phnom Penh, indicating another potential bias.

Furthermore, the introduction of an information sheet during the informed consent procedure most likely made participants more reluctant to share socially undesirable concerns and to accept audio recording. This needs to be understood in the light of the high standing and formality written communication has in Khmer society and recent privacy concerns, including the controversy surrounding a secretly audiotaped politician.^55^ In addition, the community has limited previous exposure to research and limited awareness on its purpose. A final limitation is the absence of an in-depth analysis of structural factors such as the economic and social burden of disease underlying care seeking and disease transmission dynamics.

The strength of the present study lies in the triangulation^56^ of different data collection methods and the variety of study participant profiles with a combined hospital-based and community-focused sampling strategy. In addition, this is the first qualitative study on enteric fever in Southeast Asia, and one of only few ethnographic studies on enteric fever conducted worldwide. Further research should focus on the structural constraints faced by enteric fever patients and those who diagnose and treat the disease in the community.

## CONCLUSION

The present study provides important contextual data on local perceptions and practices regarding enteric fever in Phnom Penh, Cambodia. Despite economic growth and policies put in place by the Government, health-care–seeking behavior and therapy choice for enteric fever was found to be mainly guided by economic motives. There is an urgent need to address health literacy concerning the transmission of enteric fever and food hygiene in general. Health programs could build upon preexisting and culturally defined concepts of disease causation and transmission. Future efforts to improve the uptake of appropriate diagnostics and treatment of enteric fever should take into consideration the pluralism and associated dynamics of the Cambodian health-care system.

## Footnotes

* Ofloxacin, ciprofloxacin, co-trimoxazole, ceftriaxone and gentamycin are different types of antibiotics. Ofloxacin and ciprofloxacin belong to the same group of antibiotics, namely the fluoroquinolones.
